# Characterization of the Toll-like Receptor Expression Profile in Human Multiple Myeloma Cells

**DOI:** 10.1371/journal.pone.0060671

**Published:** 2013-04-08

**Authors:** Jahangir Abdi, Tuna Mutis, Johan Garssen, Frank Redegeld

**Affiliations:** 1 Division of Pharmacology, Utrecht Institute for Pharmaceutical Sciences, Faculty of Science, Utrecht University, Utrecht, The Netherlands; 2 Department of Clinical Chemistry & Hematology, University Medical Center Utrecht, Utrecht, The Netherlands; Carl-Gustav Carus Technical University-Dresden, Germany

## Abstract

Expression and function of Toll-like receptors (TLRs) in multiple myeloma (MM) has recently become the focus of several studies. Knowledge of expression and biology of these receptors in MM will provide us with a new insight into the role of an inflammatory environment in disease progression or pathogenesis of MM. However, to date a quite heterogeneous expression pattern of TLRs in MM particularly at gene level has been described while information on the TLR expression at the protein level is largely unavailable. In this study, we investigated the TLR expression in human myeloma cell lines (HMCLs) Fravel, L363, UM6, UM9, OPM1, OPM2, U266, RPMI 8226, XG1, and NCI H929 and primary cells from MM patients at both mRNA and protein level (western blot and flow cytometry). We found that all cell lines and primary cells expressed TLR1, TLR3, TLR4, TLR7, TLR8, and TLR9 mRNA and protein. TLR2 and TLR5 were expressed by the majority of HMCLs at mRNA but were not detectable at protein level, while primary samples showed a low level of TLR2, TLR3 and TLR5 protein expression. Our results indicate that MM cells express a broad range of TLRs with a degree of disparity between gene and protein expression pattern. The clear expression of TLRs in MM cells indicates a propensity for responding to tumor-induced inflammatory signals, which seem inevitable in the MM bone marrow environment.

## Introduction

Multiple myeloma (MM) is a lymphoid neoplasm characterized by infiltration in the bone marrow of malignant plasma cells [1]. The presence of monoclonal immunoglobulins and defective innate or adaptive immune responses render MM patients vulnerable to infectious or inflammatory conditions, and in most cases these complications hamper the therapeutic approaches [2]–[4]. Furthermore, a history of infectious and chronic inflammatory diseases has been reported in certain MM patients [5]. Thus, contribution of inflammatory or infectious conditions to MM pathogenesis or progression seems plausible; however, the underlying molecular mechanisms have not been clearly deciphered. Indeed the link between inflammation and malignant conditions has long been pursued by many researchers [6]–[9]. In recent years, Toll-like receptors (TLRs), which are instrumental in integrating the innate and adaptive immune responses, have been addressed as the potential linking elements. These receptors have been detected in many cancer cells with various functional responses following their triggering. In MM, TLRs have been reported to be expressed heterogeneously on freshly isolated myeloma cells and MM cell lines, and their expression is significantly higher than on normal plasma cells[10]–[15]. However, most of the analyses have been limited to mRNA level showing inconsistencies in TLR patterns expressed by MM cells and the cellular responses following their triggering. Consequently, information on the functional protein expression patterns of these molecules is limited. Here, we present a comprehensive study on the expression profile of TLRs on established and commonly used human myeloma cell lines (HMCLs) and MM primary cells. We show strong expression of TLRs in primary MM cells as well as in all MM cell lines, which indicates a propensity for responding to tumor-induced inflammatory signals, which seem inevitable in the MM bone marrow environment.

## Materials and Methods

### Reagents and Antibodies

All the antibodies used in this study for TLR detection were from IMGENEX (San Diego, CA, USA): TLR1 (IMG-5012), TLR2 (IMG-416A), TLR3 (IMG-315A), TLR4 (IMG-5031A), TLR5 (IMG-663A), TLR7 (IMG-581A), TLR8 (IMG-321A) and TLR9 (IMG-305E). The following secondary antibodies and isotype controls were used in the FACS experiments: F(ab’)anti-rabbit IgG-FITC, anti-mouse IgG-FITC, mouse IgG2b, κ, all from eBioscience, mouse IgG2a, κ from Biolegend (San Diego, CA, USA) and rabbit normal immunoglobulin from Santa Cruz Biotechnology (Santa Cruz, CA, USA). Anti-beta actin and the following secondary antibodies used for blotting experiments were from Santa Cruz Biotechnology: horseradish peroxidase-conjugated goat anti-mouse IgG and goat anti-rabbit IgG. HRP-conjugated goat anti-rabbit immunoglobulin was from DAKO (DK-2600 Glostrup, Denmark). Monoclonal anti-human CD138-APC was from Biolegend. All PCR and cDNA synthesis reagents including Platinum® *Taq* DNA polymerase were from Invitrogen.

### Cells and Cell Culture

Human multiple myeloma cell lines, Fravel, L363, OPM-1, OPM-2, U266, RPMI-8226, XG1 and NCI-H929 were obtained from American Type Culture Collection (Manassas, VA, USA). UM-6 and UM-9 had been established by the Department of Clinical Chemistry & Hematology, University Medical Center Utrecht, Utrecht, the Netherlands [16], [17]. All the cell lines were maintained in RPMI-1640 culture medium containing 2-mM L-glutamine supplemented with 5 or 10% fetal bovine serum and intermittently with antibiotics, in a 37°C incubator with 5% CO_2_. XG1 and UM6 cell lines were cultured with 1 ng/mL and 5 ng/mL of recombinant human IL-6 (from eBioscience, San Diego, CA, USA), respectively. NCI-H929 cell line was cultured in the presence of 1 mM sodium pyruvate and 50 µM 2-mercaptoethanol. The cell cultures were within five to ten passages after thawing for the expression experiments.

Bone marrow mononuclear cell (BMNC) samples from 3 MM patients were thawed from frozen stocks. BMNC samples were surplus material from bone marrow isolated for diagnostic procedures. All patients approved use of surplus material for scientific purposes by informed consent. Use of surplus material has been discussed with and approved by the review board of the University Medical Center Utrecht. The samples were first suspended in fresh RPMI medium and kept in an incubator for a few hours. Cellular debris were removed by using Ficoll hypaque centrifugation, and rest of the samples were cultured overnight in RPMI medium supplemented with 10% fetal bovine serum, 100U/ml penicillin and 100 µg/ml streptomycin in a 37°C incubator with 5% CO_2_.

### Reverse Transcriptase-polymerase Chain Reaction

Total RNA was extracted from the cells by RNeasyMinikit (Qiagen, Valencia, CA, USA) following manufacturer’s instructions. For cDNA first strand synthesis, 2.5 µg of total RNA was reverse transcribed using SuperScript^III^ reverse transcriptase. The primer sequences used for TLR 1, 2, 4, 5, 7, 8 were described before [12].

Other primer sequences obtained from Isogen (De Meern, the Netherlands) were as follows: TLR3-forward: 5′-TGCCGTCTATTTGCCACACACT-3′,

TLR3-reverse: 5′-CAGGTGGCTGCAGTCAGCAA-3′,

TLR9-forward: 5′-TCATGACTGTGCCTGCGCTG-3′,

TLR9-reverse: 5′-AGGCGCCAGTTTGACGATGC-3′, beta-actin primers had also been already described [18]. To perform the reactions in a total volume of 25 µl, we used the following conditions: primary denaturation at 95°C for 3 minutes, 35 cycles of 30 sec at 94°C, 40 sec at 58°C, 40 sec at 72°C, and 10 minutes at 72°C.

### Quantitative Real Time Polymerase Chain Reaction

Quantitative PCR was performed for the expression of TLR3 mRNA in Fravel, L363 and NCI- H929. 500 ng total RNA from indicated cell lines was transcribed using a first strand synthesis kit (Qiagen). Two microliters from each sample was applied to SYBR green real time PCR using primers which were as described previously [19]. The beta actin primer was described above. Standard curves for targets and beta actin genes were created with two-fold dilutions of a measured concentration of pooled cDNA. The amount of amplicon for each gene (TLR3 and TLR5) in samples was then determined using log of concentration and slope of the curve and normalized to that of actin. We used a two-step PCR protocol with the following temperature profile: 5 min 95°C, 40 cycles of (15 sec 95°C, 30 sec 58 or 65°C), including a melting curve at the end.

### Western Blotting

Five to ten million cells from each cell line were harvested, washed in cold PBS and suspended in lysis buffer (150 mM NaCl, 1% IGEPAL (Sigma), 50 mM Tris, pH 8.0) followed by addition of protease inhibitor cocktail (Complete Mini pills, Roche). The lysates were then left on ice for 30 minutes, spun at 10,000×g for 10 minutes and supernatants were isolated. The protein concentration of lysates was determined with a BCA kit (Pierce) and 30 µg total protein from each lysate was electrophoresed on an 8 or 12% SDS-PAGE gel and subsequently electro blotted onto a PVDF membrane. The membranes were incubated with primary antibodies (1–3 µg) overnight at 4°C followed by incubation with HRP-conjugated secondary antibodies (1∶2000–1∶5000). The signals were detected by using ECL PLUS or ECL Prime (Amersham). Membranes were reprobed for a maximal of 3 times with different TLR antibodies and anti-beta-actin, after stripping the membranes with Restore Western Blot Stripping buffer (Pierce).

### Flow Cytometry

In FACS experiments, indirect (intra)cellular staining was performed using TLR-specific antibodies. Briefly, 10^5^ cells from each cell line or BMNCs were harvested and washed in FACS buffer, PBS+0.5% BSA+0.01% sodium azide, pelleted and suspended in permeabilization/fixation buffer (from eBioscience), and left for 20 minutes at room temperature. The cells were then washed in permeabilization buffer, pelleted and incubated with 1–2 µg of indicated primary antibody at room temperature (or 4°C) for one hour. FITC-conjugated secondary antibodies, anti-rabbit IgG and anti-mouse IgG, were added at the next step. Finally, the samples were washed, suspended in FACS buffer and then analyzed using a FACSCanto™ II flow cytometer (BD Biosciences) for TLR detection, and captured data of gated live populations were analyzed using CellQuest software. Human peripheral blood mononuclear cells (PBMCs) isolated from buffy coat (Sanquin, the Netherlands) were used as positive control in some experiments. Patient BMNCs were washed and stained with APC-conjugated anti-human CD138 in FACS buffer followed by staining with TLR-specific antibodies containing as described above. FACS analysis for TLR expression was performed on live gated CD138-positive cells.

## Results

### Analysis of TLR mRNA Expression in HMCLs

**Figure 1:**
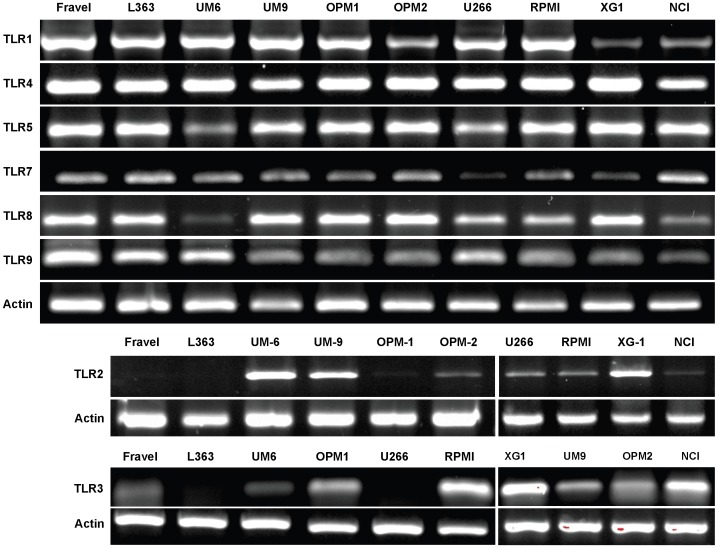
Expression of TLR mRNA in HMCLs. **A)** All the cell lines expressed mRNA for TLR1, TLR4, TLR5, TLR7, TLR8, TLR9. **B)** TLR2 mRNA was detected in all cell lines except L363 and Fravel. TLR3 showed varied expression levels in HMCLs.

Expression of mRNA for TLR1, TLR3, TLR4, TLR5, TLR7, TLR8, and TLR9 was found in all myeloma cell, but levels of mRNA expression differed amongst different cell lines (Fig. 1). TLR2 mRNA was not detected in Fravel, and L363. Expression of TLR3 mRNA was also analyzed using qPCR in 3 cell lines: Fravel, L363 and NCI-H929. The TLR3/actin expression of Fravel, L363, and NCI-H929 was 0.083, 0.043 and 0.086, respectively. Absolute quantities of TLR3 amplicon were 4.84±1.82 ng, 1.35±0.66 ng and 4.81±1.8, respectively.

### Analysis of TLR Protein Expression in HMCLs using Western Blot

**Figure 2:**
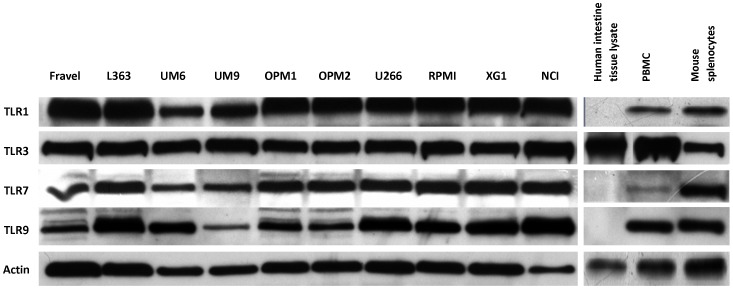
Expression analysis of TLR protein in HMCLs by Western blotting. All cell lines displayed a strong expression of TLR1, TLR3, TLR4, TLR7, TLR8, TLR9 proteins. Cell lysates were electrophoresed and blotted to PVDF membrane, which was probed with TLR-specific antibodies. To confirm the immunoreactivity of the antibodies, different positive controls were included. Beta-actin was served as loading control and was used to normalize expression levels between cells (see Figure 5). Data are representative for analysis of ≥2 independent experiments.

**Figure 3:**
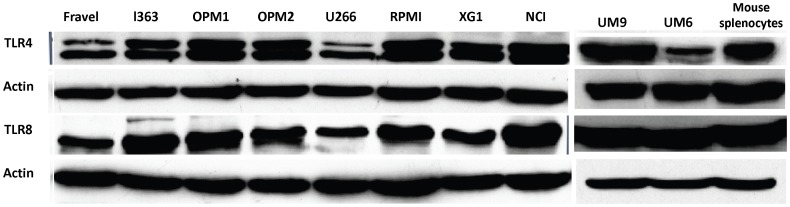
Expression analysis of TLR4 and TLR8 protein in HMCLs by Western blotting. Lysates of human intestinal tissue (A) and mouse splenocytes (B) were used as positive controls. Beta-actin was served as loading control and was used to normalize expression levels between cells (see Figure 5). Data are representative for analysis of ≥2 independent experiments.

**Figure 4:**
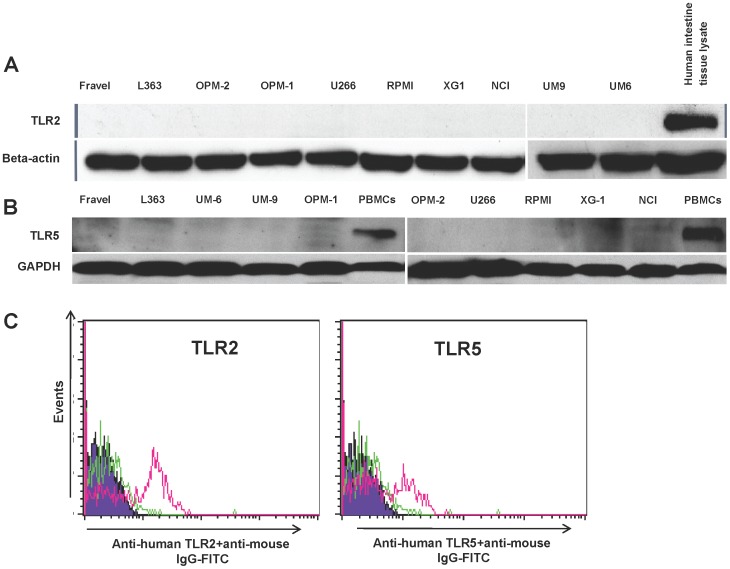
HCML do not express of TLR2 and TLR5. Expression of TLR2 and 5 at protein level was determined by western blotting (panel A and B) and FACS (panel C). The immunoreactivity of the anti-TLR2 and -5 antibodies in western blotting and FACS analysis was confirmed with human intestinal lysate (panel A and B) and human PMBCs as positive controls (panel C), respectively. Data are representative for analysis of ≥2 independent experiments.

**Figure 5:**
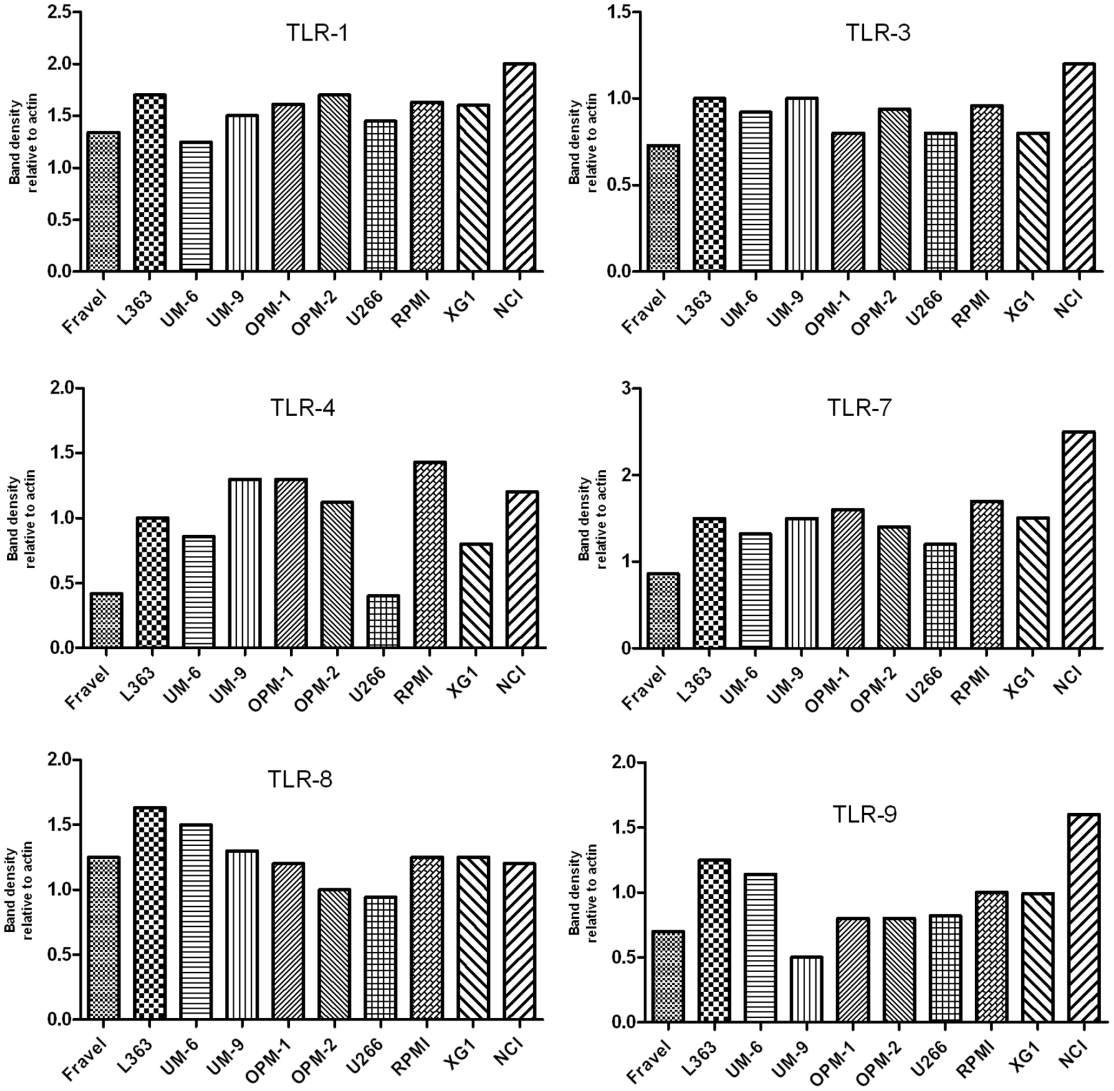
Expression of TLR1, TLR3, TLR4, TLR7, TLR8, and TLR9 for HMCLs. The expression was calculated as densitometric ratio of TLR to actin as determined by immunoblotting.

In all cell lines, expression of TLR1, TLR3, TLR4, TLR7, TLR8, and TLR9 was detected using immunoblotting (Figs 2,3). On the other hand, TLR2 and TLR5 proteins were not found in any of cell lines. The absence of signal was not due to a lack of reactivity of the antibodies used, because strong expression of these TLRs was found in human intestine tissue lysate (Fig. 4). Furthermore, using densitometry the ratio of TLR protein densities to those of beta actin was determined and showed that relative expression levels of TLRs varied amongst different cell lines (Fig. 5). For example, in TLR4 group, a prominent difference in relative protein level is obvious between RPMI 8226, Fravel and U266 cell lines. In the TLR8 group, L363 displays the highest level of the protein, and in TLR1, TLR3, TLR7, TLR9 groups the highest level of the relevant proteins is seen in the NCI-H929 cell line.

### Analysis of TLR Expression using Flow Cytometry

**Figure 6:**
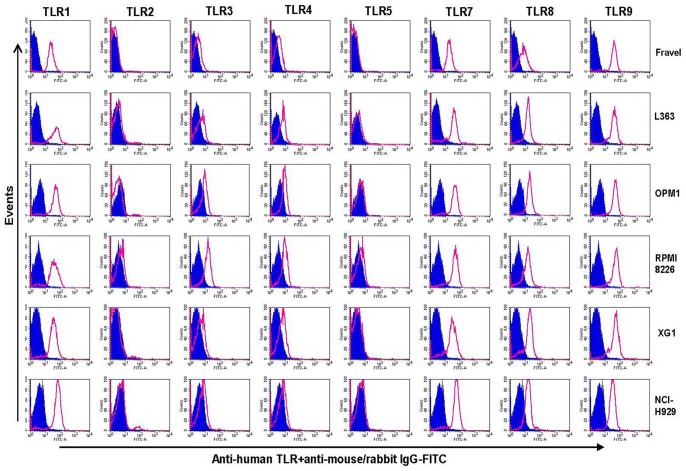
Expression of TLRs in Fravel, L363, OPM1, RPMI8226, XG1, and NCI-H929 as determined by flow cytometry. HCMLs were stained using an intracellular staining protocol with TLR-specific antibodies followed by relevant secondary fluorescent-conjugated antibodies. TLR2 and TLR5 proteins were not detectable in any of cell lines. Filled histograms (purple) represent the isotype controls and the open histograms (red) indicate specific antibodies. Data are representative for analysis of ≥2 independent experiments.

**Figure 7:**
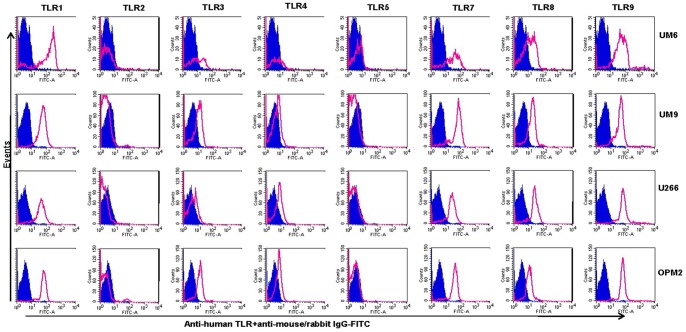
Expression of TLRs in UM6, UM9, U266, and OPM2 as determined by flow cytometry. HMCLs were stained using an intracellular staining protocol with TLR-specific antibodies followed by relevant secondary fluorescent-conjugated antibodies. TLR2 and TLR5 were not expressed by any of cell lines, confirmed also by using positive controls (see Fig. 4). Filled histograms (purple) represent the isotype controls and the open histograms (red) indicate TLR-specific antibodies. Data are representative for analysis of ≥2 independent experiments.

In the next experiments, the expression of TLR protein was analyzed by flow cytometry. TLR1, TLR7, TLR8, TLR9 were strongly expressed by HMCLs (Figs 6 and 7). In agreement with the Western blotting analysis, TLR2 and TLR5 were found not expressed by any of HMCLs in FACS analysis. In control experiments, we confirmed that antibodies used were able to detect TLR2 and TLR5 expression in human peripheral blood mononuclear cells (Fig. 4). Interestingly, expression of TLR-3 and TLR-4 in some cell lines (Fravel and NCI-H929) was very low, while the bands of these proteins in immunoblotting analysis were prominent, probably suggesting posttranslational modifications.

**Figure 8:**
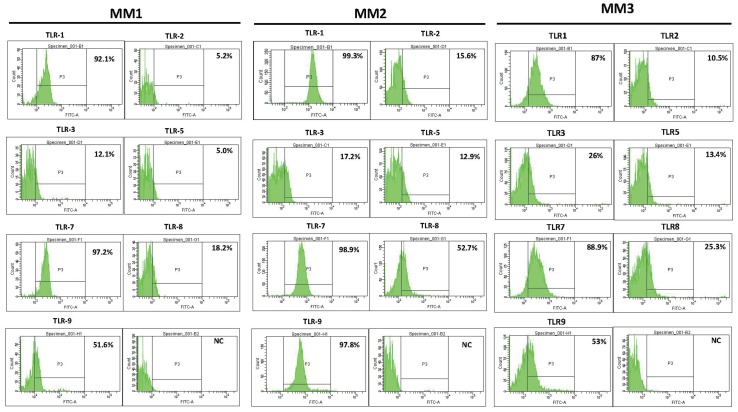
TLR protein expression pattern in primary BMNCs from 3 MM patients analyzed by flow cytometry. CD138-positive cells were gated from the total cell population. Staining with specific antibodies for TLR1, TLR2, TLR3, TLR5, TLR7, TLR8, and TLR9 was compared with isotype-matched controls (NC).

For primary MM cells, in BMNCs, live CD138-positive cells were gated and the percentage of cells binding to TLR-specific antibodies was calculated (Fig. 8). CD138^+^-BMNCs displayed a strong expression of TLR1, TLR7, TLR8 and TLR9. Some variation in the expression of TLR8 and TLR9 was found in samples of different patients. The percentage of TLR9 positive cells in MM1, MM2 and MM3 was 47.2, 97.8, and 53, respectively, while 16.7%, 52.7% and 20.8% of cells were positive for TLR8 in these patients.

## Discussion

The expression of TLRs on cells of B-lymphoid malignancies, MM and CLL, has been documented in different recent studies [10], [12]–[15], [20]. These studies, however, show inconsistencies in TLR patterns expressed by MM cells and the cellular responses following their triggering. This is the first study in which TLR expression in different HMCLs and primary MM cells has been evaluated at mRNA (RT-PCR) and protein (Western blot and flow cytometric analysis) level (Table 1). All analyzed HMCLs express mRNA for TLR1, TLR3, TLR4, TLR5, TLR7, TLR8, and TLR9. Although RT-PCR analysis is not a quantitative method, analysis of TLR3 expression in some HMCLs with realtime PCR provided a comparable expression profile. The pattern of TLR1, TLR2, TLR7, and TLR9 mRNA expression for NCI-H929, XG1, RPMI 8226, and L363 is in agreement with that described by Jego *et al* [12]. However, our results show differences with those obtained by Bohnhorst *et al* for OPM2, RPMI 8226, NCI-H929 and U266 cell lines [14]. While the pattern of TLR1, TLR2, TLR5, TLR9 mRNA expression is the same as in our cell lines, no mRNA for TLR3, TLR7, TLR8 in OPM-2, and TLR4, TLR7 in U266 cells was found in their study [14].

**Table 1:** Expression of toll-like receptors in myeloma cell lines and primary bone marrow mononuclear cells from MM patients.

	TLR-1			TLR-2			TLR-3			TLR-4			TLR-5			TLR-7			TLR-8			TLR-9		
cell line	PCR	FACS	WB	PCR	FACS	WB	PCR	FACS	WB	PCR	FACS	WB	PCR	FACS	WB	PCR	FACS	WB	PCR	FACS	WB	PCR	FACS	WB
Fravel	+	+	+	−	−	−	w+	w+	+	+	w+	+	+	−	−	+	+	+	+	+	+	+	+	+
L363	+	+	+	−	−	−	−	+	+	+	+	+	+	−	−	+	+	+	+	+	+	+	+	+
UM-6	+	+	+	+	−	−	w+	+	+	+	w+	+	+	−	−	+	+	+	+	+	+	+	+	+
UM-9	+	+	+	+	−	−	+	+	+	+	w+	+	+	−	−	+	+	+	+	+	+	+	+	+
OPM-1	+	+	+	−	−	−	+	+	+	+	+	+	+	−	−	+	+	+	+	+	+	+	+	+
OPM-2	+	+	+	+	−	−	w+	+	+	+	+	+	+	−	−	+	+	+	+	+	+	+	+	+
U266	+	+	+	+	−	−	−	w+	+	+	+	+	+	−	−	+	+	+	+	+	+	+	+	+
RPMI	+	+	+	+	−	−	+	+	+	+	+	+	+	−	−	+	+	+	+	+	+	+	+	+
XG-1	+	+	+	+	−	−	+	w+	+	+	+	+	+	−	−	+	+	+	+	+	+	+	+	+
NCI-H929	+	+	+	w+	−	−	+	w+	+	+	w+	+	+	−	−	+	+	+	+	+	+	+	+	+
**primary cells**																								
BMNC	nd	+	nd	nd	w+	nd	nd	w+	nd	nd	nd	nd	nd	w+	nd	nd	+	nd	nd	+	nd	nd	+	nd

+: positive; w+: weakly positive; −: negative; nd: not determined.

BMNC: CD138-positive cells gated from bone marrow mononuclear cells derived from MM patients (3 patients).

Analysis of TLR expression at protein level showed that TLR1, TLR3, TLR4, TLR7, TLR8, and TLR9, were expressed in most HCMLs. TLR analysis using western blotting closely correlated with the expression pattern found by flow cytometric analysis. Comparison of TLR expression at transcriptional and translation level showed discrepancies between presence of TLR mRNA and protein. For example, some cell lines expressed very low levels of TLR3 mRNA (e.g. Fravel, L363, and U266), while TLR3 protein was clearly expressed. On the other hand, presence of mRNA did not predict the expression of functional protein for some TLRs. Most notably was the marked presence of TLR5 mRNA in all HMCLs, while no expression of TLR5 at protein level was detected. This discordant relation between mRNA and protein expression may be caused by a low stability of the specific mRNA and translation and post-translational modifications of the specific protein. Similarly, Arvaniti *et al*. found that some B-CLL cells do not express TLR6 protein in spite of a high mRNA level, and also most samples display a high expression of proteins for TLR2 and TLR8 in spite of a low mRNA [21].

Expression of TLR1, TLR7, TLR8, and TLR9 in primary cells from MM patients was comparable with the profile in HMCLs, although some variation between patients was found in the extent of TLR8 and TLR9 expression. Primary MM cells showed a low level of TLR2, TLR3 and TLR5 expression as compared to HMCLs, while in all 10 HMCLs a strong signal for TLR-3 but no expression of TLR2 and TLR5 was found. Such heterogeneity in MM TLR expression and the observed differences between HMCLs and MM primary cells has also been described in recent studies. The pattern of TLR gene expression in MM cells is strikingly different from normal bone marrow plasma cells [14] or normal B cells [10]. For instance, TLR2, TLR3, TLR4, TLR5, TLR8 genes are not expressed in normal B cells [20], but expressed by most HMCLs as shown in our study and others [12], [14], or in MM primary cells [22]. This difference may be attributed to the malignant transformation of B cells during MM oncogenic alterations. Others have found similar changes in TLR expression when normal peripheral blood plasma cells are compared to normal B cells [19]. This may also suggest that the origin of the tissue may have determined the TLR expression pattern. Of note, mRNA for TLR3, TLR4, and TLR8 was not detected in B cells of B-CLL patients [23], implying that MM cells may differently regulate the expression of specific TLRs.

Taken together, our expression analyses indicate that HMCLs display a broad range of TLRs at gene and protein levels. This study also shows that analysis of mRNA alone may not provide a correct prediction of functional TLR protein expression in HMCLs [24]. Indeed, strong expression of TLRs in HMCLs and primary tumor cells indicates a propensity for responding to tumor-induced inflammatory signals which seem inevitable in MM bone marrow environment. TLR triggering on HMCL and MM primary cells has been associated with heterogeneous effects including increase in proliferation, survival, cytokine and chemokine production, induction of apoptosis or protection from apoptosis, drug-resistance and immune escape [11]. Our study suggests that effects on tumor cells through stimulation of TLRs by endogenous ligands such as soluble syndecan-1, matrix metalloproteinase products, heat-shock proteins, HMGB-1, which are released due to cell necrosis, should also be considered in MM [25].
